# Post-disaster research: Is there gold worth the rush?

**DOI:** 10.4102/jamba.v7i1.120

**Published:** 2015-04-30

**Authors:** JC Gaillard, Christopher Gomez

**Affiliations:** 1 School of Environment, University of Auckland, New Zealand; 2 Department of Geography, University of Canterbury, New Zealand

## Introduction

Dynes, Haas and Quarantelli (1967) once set the agenda for disaster research as follows:

high priority is given to those disasters which are quick and unexpected, which affect more than one industrial community, where there is heavy property damage, where the number of casualties exceeds 100 and which elicits the participation of national organizations during the emergency period. (p. 46)

Almost 50 years afterwards, major disasters continue to stir the prime interest of researchers, who often immediately rush to the affected areas to conduct studies of various kinds, from hazards observations to social surveys on the impact of the events and post-traumatic stress disorder research. Stallings (2007:56) actually suggests that ‘arriving on site as soon as possible is generally seen by field researchers as key to the success of their work’. Recently, this ‘research gold rush’ has been observed in the regions hit by the 2004 Indian Ocean tsunami, Hurricane Katrina in the United States of America (USA) in 2005, the 2008 earthquake in China, the 2010 earthquake in Haiti, the 2010–2011 Canterbury earthquakes in New Zealand and the 2011 earthquake and tsunami in Japan.

**FIGURE 1:**
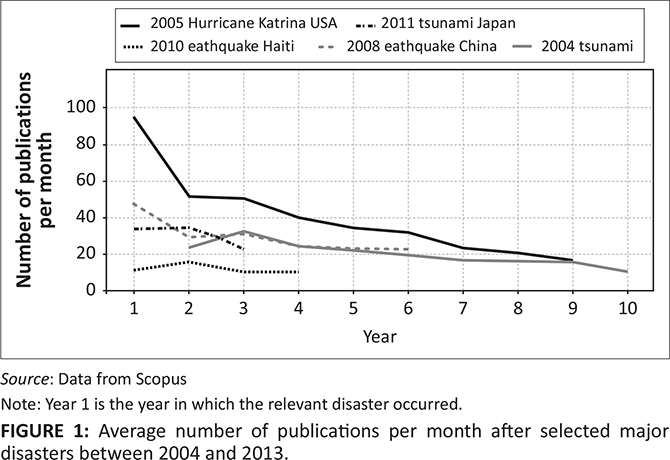
Average number of publications per month after selected major disasters between 2004 and 2013.

*Source*: Data from Scopus

Note: Year 1 is the year in which the relevant disaster occurred.

A quick analysis of academic peer-reviewed articles related to the foregoing events (which have stimulated the highest academic attention over the past 15 years) available from Scopus shows that the number of publications peaked immediately or a year after the disasters (Figure 1). This is particularly evident for Hurricane Katrina, which has been the focus of more than 3500 peer-reviewed publications, including 382 before the end of 2005.

Of course, not all these quick post-disaster publications have required field work and immediate field studies, but many have. Although most researchers engage in such research for laudable reasons, little reflection has been given to the implications and ethics of such practice. The present commentary aims at opening up a debate around these.

## Temptation and opportunity

Rushing to affected areas immediately after the event is very tempting for researchers interested in disasters. What White and Haas (1975) called ‘post-audits’ have indeed long been deemed essential for better understanding the impact of natural hazards as well as people's response to the events, and, in consequence, for enhancing policies for disaster risk reduction (DRR) (e.g. Killian 1956; Mileti 1987; Stallings 2002, 2007). Quarantelli (1997) provides two basic reasons why it is allegedly so important to get to the scene as soon as possible after the event:

first, observations can be made and documents can be collected that cannot be obtained through later interviewing. The social barriers that normally exist to restrict access to high level officials and key organizations, simply to not exist. Second, being on the scene early insures a high degree of access and cooperation. Victims are typically candid, cooperative and willing to talk in ways far more difficult to get later. (p. 57)

Stallings (2007:61) further adds that eventually ‘respondents’ personal recall that may be skewed by repeated retelling of their stories to a succession of interviewers’. Researchers who rush to disaster-affected areas thus justify their approach by the perishable nature of the data they need to collect (Bourque, Shoaf & Nguyen 1997).

Although the collection of perishable data is often essential, both for the sake of the local affected and the international community, the multiplication of initiatives from different countries and research groups sends a very large number of individuals to the impacted areas. For example, in the immediate aftermath of the 2004 tsunami, teams of physical and social scientists from France, Japan, Russia and the USA – to cite just a few – went through a data collection exercise in Indonesia with little or no coordination at first (e.g. Borrero 2005; Kawata *et al*. 2005; Iemura *et al.* 2006; Moore *et al.* 2006; Lavigne & Paris 2011); some suggested that Aceh was actually ‘ransacked’ by researchers (Missbach 2011).

Moreover, the disaster gold rush often although not always sees the convergence of researchers with limited knowledge of the area as well as insufficient time to conduct prior appropriate literature review – a problem already mentioned by Killian (1956) 60 years ago, and that can lead to misconceptions (Gomez & Hart 2013). This raises the question of scientific validity and quality, but also that of the drivers at play in the background. It should indeed be asked ‘whose needs drive the process of inquiry and data gathering?’ (Schein 1987:32).

The ‘gold rush attitude’ is undoubtedly the product of realms of the contemporary scientific scene where researchers need to produce a large number of publications every year in scientific journals, and are being ‘measured’ using the h-index, m-index, etc. (Eloy *et al.* 2014). This behaviour is first encouraged by the journals themselves, as the first publications will be cited by the next wave of publications. Being the first to conduct research in the aftermath of a disaster thus provides a ‘competitive advantage’, in the words of Stallings (2007:61), on the ‘publication market’. Publishing as much and as soon as possible after a disaster is also encouraged by the researchers’ own institutions, as the volume of publications increasingly becomes a guarantee of ‘excellence’ (Danell 2011) and often a requirement for a continuing employment, as Cupples (2011:338) pointed out, for neoliberal campuses. The above-mentioned chain of pressure is even longer, as universities are in turn attracted by the ‘carrot’ of the world universities’ rankings.

It is therefore very difficult for the individual researcher not to be instrumental in the international institutional competition, even if one tries to act within a strict ethos. One could even argue that it is more the chain that links the world of academic rankings to the disaster-impacted communities that needs to be broken.

Although it would be presumptuous to pretend to provide a solution to this issue, one would wonder if a regionalisation of reconnaissance teams and the areas they work in would not reduce the ‘gold rush’ issue. The all-important data that needs collection could therefore be collected by a smaller number, before being shared for the benefit of the international community.

## Rushing to the unknown for the best … or not?

Even in the face of intense competition amongst academics, mostly fuelled by the international education business, the authors recognise that such research has emerged from a real desire to ‘do good’. The ‘gold rush’ is indeed usually motivated by a genuine will to contribute to the recovery/DRR effort by enhancing our understanding of the event. Again, this is a fair and commendable objective for researchers familiar with both the place and the local culture; their pre-existing networks of stakeholders and friends, including those directly affected, may allow them to collaborate in post-disaster recovery or DRR in continuity of their previous engagements.

Nonetheless, one may wonder whether it is appropriate for outsiders less familiar with the affected places, who may lack prior cultural and language skills, to converge upon places where people are struggling to rebuild their lives and livelihoods, and have other priorities than answering questions about the recent events (Schenk 2013). Ethically these outside researchers may sit on the fence, as suggested in the larger field of development studies (Cooke 2004; Sidaway 1992).

If post-disaster ethics accepts behaviours that would not be allowed during ‘normal’ or pre-disaster times, the controversial actions are all answering an imperative of immediate necessity and immediate ‘good’, serving either the community or the objectives set by the local leaders. The famous war-time analogy, explaining that the life of a soldier who can go back to the front is more valuable than that of the injured soldier who is not able to contribute anymore, is an excellent image of what is unacceptable during ‘normal times’ becoming acceptable during exceptional circumstances.

However, outside researchers are not part of the ‘army’: they did not see the battle. Moreover, as outsiders they most often do not contribute to the greater good or higher objectives of the community or to the goals set by the local leaders. To assess their presence from an ethical standpoint researchers still need to be integrated in the equation, as they are actors – willing or not – in the devastated places.

If one considers that the ultimate goal is the rebuilding of the place, of lives and livelihoods – that is, the greater good, or at least good for the greater number – the presence and actions of outside researchers can be evaluated in term of impacts on the local community, regardless of the researchers’ own objectives and motivations. One may then ask whether (and if so, how) researchers who need a translator, who extract data from the community, who possibly behave inadequately because of a lack of cultural understanding, and also consume local resources to sustain their own needs (obviously often different to those of people affected by the event) have a positive effect to the local rebuilding. These questions have long been asked in the broader literature on the ethics of academic fieldwork, especially in the context of researchers from wealthy countries visiting poorer countries (Desai & Potter 2006; Scheyvens & Storey 2014). However, in a post-disaster context, all of these issues are exacerbated. In such cases the presence of outside researchers shortly after a disaster may be inappropriate, as they may be detrimental to the good of the greater number. To continue with the previous analogy, the outside researchers would be the soldiers that the army should give less priority to.

## Beyond researchers’ good will: Historical heritages, power relations and perceptions

Although most researchers try to limit the negative effects of their interactions with local communities, one cannot but wonder how the goodwill is perceived by the local communities, and on what historical layer these interactions come to rest. This observation becomes particularly valid when considering the majority of researchers’ movements from centre to periphery that is, from the wealthiest to the less affluent countries, or from the most important urban centres to less significant towns and remote rural areas, mirroring colonial and postcolonial power relations amongst and within nations (Altbach 2003; Sidaway 1992).

Present-day researchers certainly cannot be blamed because they dispose of the financial potential to ‘act’, but history teaches us that research gold rushes are not a novel occurrence, and have been an important part of the West European and North American diplomatic toolbox during at least the past two centuries. It was the tool of a colonial and postcolonial era which has vehiculated ideas of hierarchy between cultures (Bankoff 2001; Escobar 1995; Said 1994). Scientific and engineered by-products were then used to enhance the power of the centre over the periphery and the power of the local elite who collaborated (Mrazek 2002). Although formal colonies are a landscape that officially ended with the 20th century, cultural and social inequalities are still embedded within centre-periphery interactions (Souza 2011). This is also the case in the academic realm, where there are obvious unequal power relations between universities and researchers of the wealthiest nations and central cities and those of the once-colonised world, hence often from the less affluent countries, as well as those from peripheral towns (Altbach 2003; Tuhiwai Smith 2012).

Furthermore, post-disaster research flurries frequently occur in dissociation from local researchers. If not, local researchers tend to accept forms of collaboration that place them in the position of assistants rather than leaders or equal collaborators (Missbach 2011), despite the fact that they are the most legitimately able to conduct and drive studies in the affected areas. This legitimacy is not only built upon their expert knowledge of the local places and people, but is also inherited from their personal relevance as they were, are and will be part of the affected areas and communities. The potential long-term and short-term positive impacts they can have may be way greater than those of any outside researchers, who may be ignorant of the local language and culture.

**FIGURE 2:**
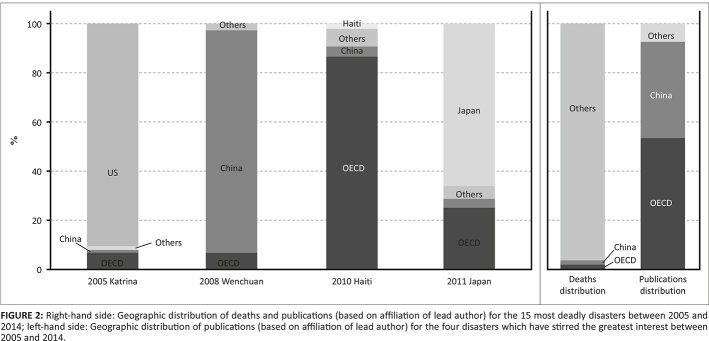
Right-hand side: Geographic distribution of deaths and publications (based on affiliation of lead author) for the 15 most deadly disasters between 2005 and 2014; left-hand side: Geographic distribution of publications (based on affiliation of lead author) for the four disasters which have stirred the greatest interest between 2005 and 2014.

A review of articles published on the 15 largest disasters in terms of number of people killed, based on data from the Center for Research on the Epidemiology on Disasters (2015), of the past 10 years, shows how unbalanced power relations between Western (i.e. in this case those from the Organisation for Economic Co-operation and Development [OECD] countries) and non-Western researchers are, with the exception of China, which can be considered a special case in the contemporary academic world (Figure 2).

Overall, more than 95% of deaths are associated with disasters which occurred in non-OECD countries, but more than 58% of the publications related to these events were led by authors based in OECD countries, according to the Scopus database. A closer look at the four events which stimulated the largest number of articles, for example, Hurricane Katrina in 2005 in the USA, the 2008 Wenchuan earthquake in China, the 2010 Haiti earthquake and the 2011 earthquake and tsunami in Japan, further emphasises that US, Japanese and Chinese researchers have a strong control over research conducted in the aftermath of disasters in their own countries. However, Haiti-based researchers have only led 2% of the publications related to the 2010 earthquake, versus almost 87% for researchers based in OECD countries.

This postcolonial background explains why the ‘goodwill’ therefore often proves to be a one-way street, and it is obviously seldom that researchers from Indonesia or Haiti (examples given for the sake of the argument) go to the USA and Japan in the aftermath of disasters to teach local researchers how to proceed.

## Major disasters and beyond

Massive and numerous research initiatives following major disasters lead scientific attention and discussion to be disproportionally focused and based upon large-scale events, to the detriment of small-scale, everyday hazards, the cumulative impact of which is yet supposed to have a larger effect on those affected (Wisner & Gaillard 2009). In fact, despite a global momentum which emphasises the hypothetical importance of these small-scale and neglected disasters (e.g. International Federation of Red Cross and Red Crescent Societies 2006; United Nations International Strategy for Disaster Reduction 2009; Global Network of Civil Society Organisations for Disaster Reduction 2013), there is a dearth of case studies which provide actual data at local level. It proves much more challenging for academics to research small-scale (and sometimes lingering) disasters, which are overlooked by the media, policy makers and practitioners of DRR altogether, because these events are difficult to grasp. If it is easy to concur that Hurricane Katrina in the USA or the earthquake which hit Haiti in 2011 led to disasters, it proves much more difficult to affirm, from the viewpoint of an outside researcher, that a small landslide affecting a couple of families in a remote region of the very same countries also results in a disaster – although it would be so in the eyes of said households.

In addition, in the present academic system it is somehow less rewarding for most researchers to focus on a series of small events, which do not hit media headlines, than to focus on major disasters upon which policy attention is focused and towards which, in consequence, research money flows. Henceforth, Dynes *et al.*’ s (1967) research agenda, set almost 50 years ago, continues to prevail and research on disasters is still largely biased towards quick and large events affecting centre (core) regions of a particular country or cities (at the detriment of rural areas) in more remote locations, for example, Banda Aceh following the 2004 tsunami in Indonesia or Tacloban in the aftermath of typhoon Yolanda in the Philippines in 2013. These are usually the easiest to access in a fashion which Chambers (1984), in the larger context of development, coined quick-and-dirty ‘tourism’ (i.e. research). The abundance of such research ultimately strengthens the so-called ‘paradigm of the extreme’, which still dominates disaster studies as well as policies for DRR, despite its obvious failure to address the root causes of the problem and thus significantly reduce the occurrence of disasters worldwide (Gaillard 2010; Hewitt 1983).

## Balancing views for a better world

The downside of post-disaster research should, however, be balanced with its more positive dimension and useful contribution, which cannot be neglected at the benefit of a romanticised perspective on the role of local knowledge and researchers. In many instances, such as in the case of the 2010 Haiti earthquake, the research capacity of the local academic community is greatly altered by the disaster, and accumulating more knowledge about the event may come as a second priority for scholars struggling to rebuild their own lives. One also needs to recognise that there are often more resources, especially with regard to equipment and gadgets for physical scientists, available in wealthy Western countries than in less affluent peripheral regions of the world. These resources are sometimes needed to advance knowledge and enhance policies and practices geared towards reducing the risk of disasters both locally and internationally.

Moreover, the views of local researchers (as much as those of Western researchers in certain instances) may be constrained by authoritarian political regimes (Sidaway 1992). This may lead local perspectives to be either biased towards the interests of the State (or any other powerful stakeholders) or kept unheard for fear of repression. Finally, a greater number of scholars from peripheral regions of the world are nowadays able to study and work in Western countries, thus opening up new opportunities for delocalised expertise following disasters.

In this context, the views of outside researchers often remain useful and valuable. In fact, excluding outside, especially Western researchers from researching major disasters may lead them to evade their own responsibility, as Radcliffe (1994:28) puts it in the context of global gender relations, ‘with regard to global relations of privilege and authority which are granted, whether we like it or not, to First World women (and men)’.

Rather, the point here is to argue for more harmonious and balanced collaborations wherein local experts would lead post-disaster research, from the early project design stage through to authorship in publications with, when needed, the support of outside researchers obedient to cultural norms and values. The latter also includes experts from places often hit by disasters, i.e. Haiti, Indonesia or the Philippines, with therefore greater experience of post-disaster research, to come and support Western colleagues when events happen in their own countries. Such a posture obviously demands a major shift in power relations, not only on the part of researchers but also on that of funding agencies and academic journal editors, amongst other stakeholders of post-disaster research, who need to express greater trust in institutions and colleagues usually struggling at the margin of the academic world.

Ultimately, as long argued by Chambers (1995) in the context of development research, it first and foremost relies upon changing attitudes and behaviours by those currently holding power. The first step towards this paradigm shift may lead to a code of ethics for post-disaster research.

## Towards a code of ethics for post-disaster research

If Killian (1956) raised some of these issues in his pioneer exploration of field studies in disasters, and Kelman (2005) more recently put forward some salient points in disaster research at large, very few academic discussions have since occurred with regard to the ethical and conceptual legitimacy of rushing to places affected by disasters in the immediate aftermath of the event for conducting various kinds of research. Psychology and biomedical sciences are the limited exceptions to the rule in the context of their narrow fields (Collogan *et al.* 2004; O’Mathúna 2010; Sumathipala *et al.* 2007). Yet the field of disaster studies at large has grown huge, and related publications have soared over the past few decades.

In parallel, the increasing attention on disasters from a growing number of media, policy makers and practitioners dealing with DRR seemingly accentuates the demand for research in a greater number of places and on more diverse and specialised topics. This is why it now seems urgent to reflect upon both the underpinning rationale for conducting field studies following disasters, and the approach to be taken to conduct such research if it is deemed necessary for diverse reasons, including for rebuilding the lives and livelihoods of those affected and reducing the risk of future disasters. These reflections seem ethically and conceptually imperative to further disaster studies in a meaningful direction.

Our hope is that these lead to an international code of ethics for post-disaster research, as there already exists for emergency physicians. It is essential that this code is built under Rawls’ (1999) ‘veil of ignorance’ to ensure that the social contract engaging the different parties is fair and just. In fact, some countries frequently affected by disasters have already moved in this direction. For example, the Philippines national ethical guidelines for health research, which also apply to behavioural and social sciences, dedicate three pages to appropriate conduct in times of emergencies and disasters (Philippine Health Research Ethics Board 2011). One may just wonder how many outside researchers presently doing research in the areas affected by typhoon Yolanda in 2013 have actually read these guidelines?
